# Book Review

**Published:** 1947

**Authors:** 


### Reviews of Books

**Centennial of Surgical Anæsthesia : an Annotated Catalogue.** By J. F. Fulton, M.D., and Madeline E. Stanton, A.B. Pp. xv., 102. Illustrated. New York: Henry Schuman. 1946. Price 24s. **A Memoir to the Academy of Sciences at Paris (in 1847) on a new use of Sulphuric Ether.** By W. T. G. Morton, Foreword by J. F. Fulton. Pp. vi., 24. New York: Henry Schuman. 1946. Price 9s.—It is fitting that the hundredth anniversary of the discovery of ether anæsthesia should arouse interest in the story. The *Centennial* gives notices, and often facsimile pages, of all the principal publications. It opens with Dioscorides and Pliny, praising the virtues of mandragora. The tract by Beddoes and Watt on " Factitious Airs," written in Bristol in 1794, is described. Humphrey Davy, working in Dowry Square, Hotwells, discovered the anæsthetic properties of nitrous oxide, using S. T. Coleridge, Robert Southey and other well-known men as his human " guinea pigs." Mesmerism had a brief vogue, then sulphuric ether, then chloroform. There is a colourful story connected with Morton's introduction into general use of sulphuric ether. He tried it first as a local agent ; then gave it by inhalation. He tried to patent it under the name of Letheon, but surgeons would not use it until the nature of the drug was declared. There was a noisy controversy about priority. Morton tells the story of his trials, failures and triumphs in his tract, concluding with the remark that he was the only man in the world who lost money out of the discovery. Anæsthesia was a venture of youth. Humphrey Davy was twenty-one, Wells twenty-three, and Morton twenty-seven. Both books are most interesting. So they should be, for they describe the greatest scientific discovery of all time.

**The Rheumatic Diseases.** Second Edition. By G. D. Kersley, M.A., M.D., F.R.C.P. Pp. xii., 120. Illustrated. London: William Heinemann, Medical Books Ltd. 1945. Price 15s.—This is a sound and interesting exposition on the rheumatic diseases, written by one who is a recognized master of his subject. In the space of little more than a hundred pages Dr. Kersley gives a concise yet full description of such conditions as rheumatic fever, rheumatoid arthritis, osteo-arthritis, spondylitis, gout, fibrositis, and sciatic pain. It is thus an excellent book both from the point of view of the student and practitioner for whom it is particularly written. After the narrow and bigoted descriptions of sciatic pain to which we have become accustomed of recent years, it is refreshing to read an account of this state in which the various causes and the theories appertaining thereto are clearly set forth without bias. Dr. Kersley states that at the Mayo Clinic, out of five thousand five hundred cases of sciatica, 13 per cent. were associated with an intervertebral disc lesion, and of these 85 per cent. are stated to have been cured by operation with a mortality of only 0.25 per cent. He adds that this percentage of cures is certainly higher than that usually observed, unless the criterion of cure is the variant factor. In the discussion on fibrositis, after describing the various ways in which this condition manifests itself in the body, he gives an excellent account of the treatment, and his words "A good masseur or masseuse is born with hands and personality" will convey a great deal to those who have had experience in treating this widespread malady. There is no question that this admirable book should find a place on the bookshelf of every up-to-date practitioner.

**Atlas of the Commoner Skin Diseases.** By HENRY C. G. SEMON, D.M., F.R.C.P., and ARNOLD MORITZ, M.B., B.C. Third Edition. Pp. viii., 343. Illustrated. Bristol: John Wright & Sons Ltd. 1946. Price 50s.—This Third Edition of Semon and Moritz's *Atlas* was prepared in the difficult closing period of the flying-bombs in London. Its high standard is fully maintained and it forms an indispensable adjunct to the various text-books of dermatology. The authors and the publishers deserve the highest credit for bringing out such a production in 1946. We have one criticism to offer on the colour reproduction, namely, that the skin-tints are greatly improved if the plates are viewed through a pale-blue glass. It may not be possible to add the blue in the process of colour-printing, but as the plates stand they tend to require this addition.

**The Causation of Appendicitis.** By A. RENDLE SHORT, M.D., B.S., B.Sc., F.R.C.S. Pp. viii., 79. Illustrated. Bristol: John Wright & Sons Ltd. 1946. Price 10s.—The cause of the commonest abdominal emergency is still to seek. In 1920 Mr. Rendle Short put forward the hypothesis that appendicitis was associated with the relatively smaller quantity of cellulose eaten in various communities since 1895. Now, at a time of life when most surgeons are content to settle down and rest on their laurels, he has set himself the task of probing the subject again and has put the results on paper in the form of a small book. The facts of the incidence of the disease are hard to come by, but he deals honestly with those available, which he has been at pains to glean from many sources. It would appear that the disease became very much more common round about 1895. This fact is corroborated from the surgical records of Guy's, St. Thomas's Hospital and the Bristol Royal Infirmary, and it is this increased incidence which he attempts to analyse and explain. The aetiology of the disease remains an enigma at the end, but it is at any rate more than salutary to hear a surgeon with such a vast experience of appendicitis thinking aloud on the subject. Where the temptation to fanciful theories is so great, he must be congratulated on his persistent honesty in dealing with the known data on the subject.

**Early Diagnosis of the Acute Abdomen.** By ZACHARY COPE, M.D., M.S., F.R.C.S. Ninth Edition. Pp. xvi., 262. Illustrated. London: Oxford University Press (Geoffrey Cumberlege). 1946. Price 12s. 6d.—The doctor who buys this book acquires indispensable surgical wisdom in a publication first-class in paper, type and pictures. He will be impressed by the necessity of coming to a conclusive opinion when first confronted with abdominal crises—a vital start for success with this major issue of practice, both general and hospital. The author shows how greatly the patient's prospects brighten by early treatment of the acute abdomen. Another fundamental, the bearing of anatomy on the symptom-complex of the various disasters receives due emphasis. Radiography is given the prominence it merits, but which, for the elucidation of acute conditions, is not generally assigned to it. Among individual problems the acute appendix is dealt with in a most masterly and complete manner, proportionate to our commonest emergency. The same is true of intestinal obstruction. Most acceptable are the comparative tables of classified symptoms and signs which help the clinician to distinguish chest and urinary diseases from the intra-peritoneal disasters that they mimic. So sound a treatise teeming with surgical maxims is a boon companion for the young clinician. Let us hope the author will still further perfect it, possibly replacing some lines on cutaneous hyperæsthesia, by the following additions: (1) Diarrhœa (*sic*) so rife in our decade, should be in bold type and have a paragraph in the appendix chapter; (2) Auscultation merits heavy type (page 51); (3) the records of the abdominal circumference in dubious intestinal obstruction should find a place; (4) E.U.A., examination under anæsthetic, as the "final court of appeal," occasionally saves the exploratory plunge with a "carving" knife, or directs it more advantageously.

**Practical Anæsthetics.** By HOEL PARRY-PRICE, M.R.C.S., L.R.C.P., D.A. Pp. viii., 127. Illustrated. Bristol: John Wright & Sons, Ltd. 1946. Price 12s. 6d.—Anæsthetics, like munitions, were produced in greater quantity during the war than in peacetime. Many Service Medical Officers, with only the slightest acquaintance with the subject, had anæsthetics thrust upon them, and a large proportion eventually became recognized as specialists. They owed much to the small number of experienced anæsthetists, spread very thinly through the Services, who had already completed their training before the war, and were able to guide the steps of their younger followers. Surgeon Commander Parry-Price was one of this small band; and his little book is written mainly for the benefit of the Sick Berth Staff and of the young anæsthetist in the Royal Navy. The book covers a wide range, and much valuable information is crowded into its small pages. This compression necessitates a somewhat patchy and dogmatic treatment, and some of the statements expressed will not command universal agreement. There are chapters on gas-oxygen and gas-oxygen-ether; cyclopropane, ether, and endotracheal anæsthesia; intravenous and local anæsthesia; anæsthetics for special operations; carbon dioxide absorption; premedicant and supportive drugs; accidents and complications; and the contrast between Service and civilian anæsthetics. The usual plates of apparatus are included, and they are supplemented by a few photographs which are not of very high quality. The book will be found of real value by theatre orderlies and young anæsthetists, and there are many practical tips which the more experienced could read with profit. Twelve shillings and sixpence, however, seems a high price to pay for so small a work.

